# Supplementary material for: Complex‐centric proteome profiling by SEC‐SWATH‐MS
Source: Mol Syst Biol. 2019 Jan 14;15(1):e8438. doi: 10.15252/msb.20188438 (PMC6346213; doi:10.15252/msb.20188438)
Supplement: Supplementary file 8 — Dataset EV7 [file MSB-15-e8438-s008.zip › feature_plots_string/Q9HAW0.pdf]

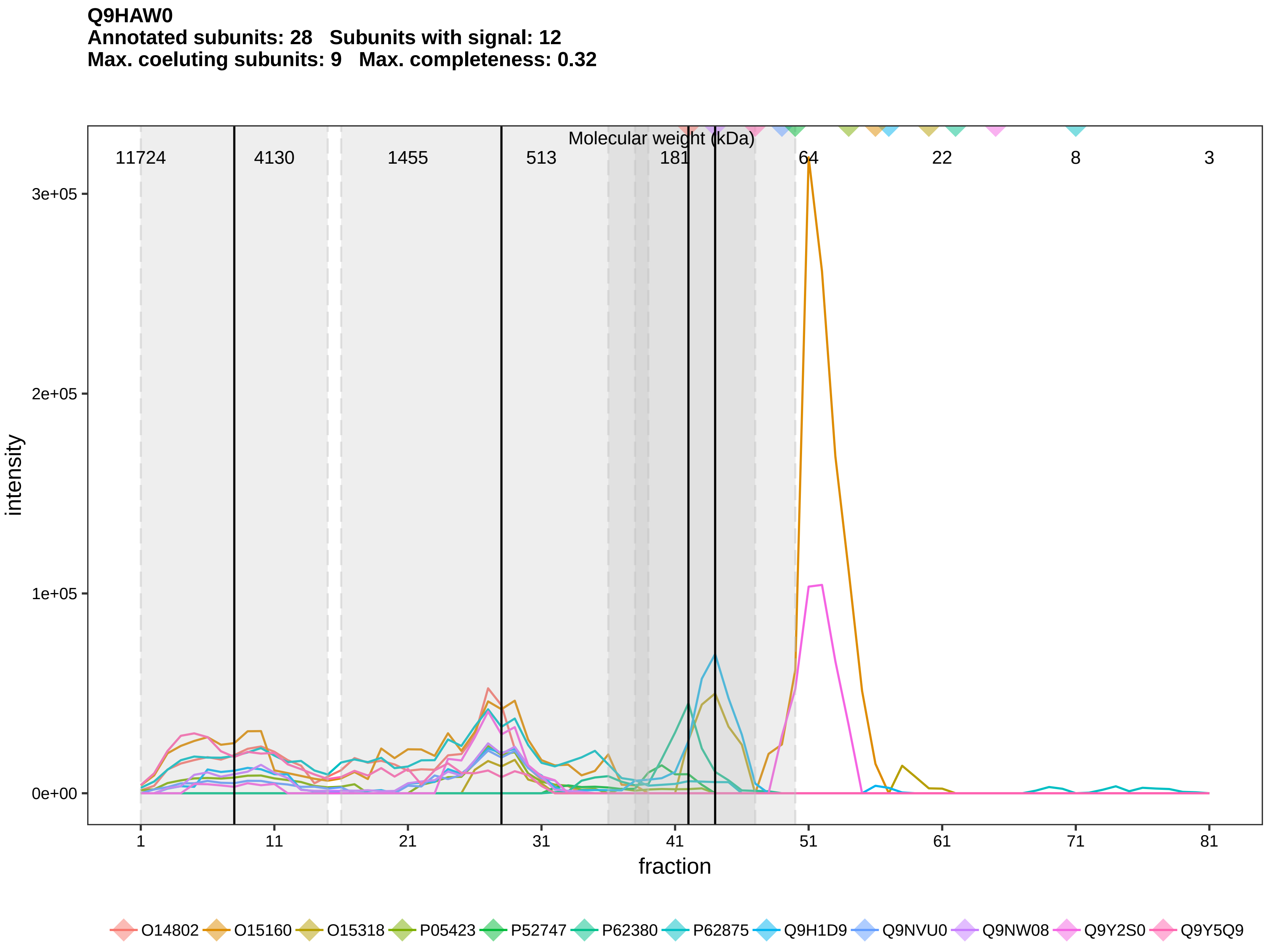
Q9HAW0
Annotated subunits: 28 Subunits with signal: 12
Max. coeluting subunits: 9 Max. completeness: 0.32
Molecular weight (kDa)
11724
4130
1455
513
181
64
22
8
3
intensity
3e+05
2e+05
1e+05
0e+00
fraction
1
11
21
31
41
51
61
71
81
O14802
O15160
O15318
P05423
P52747
P62380
P62875
Q9H1D9
Q9NVU0
Q9NW08
Q9Y2S0
Q9Y5Q9
